# Automatic Extraction of Protein Point Mutations Using a Graph Bigram Association

**DOI:** 10.1371/journal.pcbi.0030016

**Published:** 2007-02-02

**Authors:** Lawrence C Lee, Florence Horn, Fred E Cohen

**Affiliations:** 1 Department of Cellular and Molecular Pharmacology, University of California San Francisco, San Francisco, California, United States of America; 2 Biomedical Informatics, University of California San Francisco, San Francisco, California, United States of America; 3 Laboratoire de Biologie, Informatique et Mathématiques, Commissariat à l'Energie Atomique, Grenoble, France; 4 Department of Biochemistry and Biophysics, University of California San Francisco, San Francisco, California, United States of America; University of California San Diego, United States of America

## Abstract

Protein point mutations are an essential component of the evolutionary and experimental analysis of protein structure and function. While many manually curated databases attempt to index point mutations, most experimentally generated point mutations and the biological impacts of the changes are described in the peer-reviewed published literature. We describe an application, Mutation GraB (Graph Bigram), that identifies, extracts, and verifies point mutations from biomedical literature. The principal problem of point mutation extraction is to link the point mutation with its associated protein and organism of origin. Our algorithm uses a graph-based bigram traversal to identify these relevant associations and exploits the Swiss-Prot protein database to verify this information. The graph bigram method is different from other models for point mutation extraction in that it incorporates frequency and positional data of all terms in an article to drive the point mutation–protein association. Our method was tested on 589 articles describing point mutations from the G protein–coupled receptor (GPCR), tyrosine kinase, and ion channel protein families. We evaluated our graph bigram metric against a word-proximity metric for term association on datasets of full-text literature in these three different protein families. Our testing shows that the graph bigram metric achieves a higher F-measure for the GPCRs (0.79 versus 0.76), protein tyrosine kinases (0.72 versus 0.69), and ion channel transporters (0.76 versus 0.74). Importantly, in situations where more than one protein can be assigned to a point mutation and disambiguation is required, the graph bigram metric achieves a precision of 0.84 compared with the word distance metric precision of 0.73. We believe the graph bigram search metric to be a significant improvement over previous search metrics for point mutation extraction and to be applicable to text-mining application requiring the association of words.

## Author Summary

In biological research, new information is often presented in the form of peer-reviewed published journal articles. Despite the best efforts of electronic database curators, a majority of this information is still found only in textual form, and thus excluded from direct computational analysis. One such type of information that is abundant in scientific literature is protein point mutations. We seek to extract protein point mutation examples from the literature and to associate them with a unique protein name and species of origin in a standardized protein database. To do this, we have created an application that searches for and retrieves full-text articles from publishers, identifies point mutation terms, protein name terms, and organism name terms within the articles. We describe Mutation GraB, an application that utilizes a graph shortest-distance search in concert with word bigram analysis that is used to find significant associations between these terms in the text. This graph bigram search metric was found to be reasonably effective at identifying correct protein point mutation pairs and represents a good compromise between accuracy and broad applicability. The application can be applied to a large set of journal literature from a protein family to generate a database of point mutations.

## Introduction

With the advent of ultra high–throughput screening and high-density array technology, the biological community has come to appreciate the value of unbiased surveys of complex biological systems. Bioinformatics tools have become an integral part of the analysis of these extensive datasets. When complex data is collected centrally, the analysis can be straightforward. When data is collected in a distributed fashion, investigators must agree on a centralized data-deposition strategy or we must develop tools to interrogate the published literature and extract relevant information. Manually curated online databases have developed to meet this need, but they are difficult to maintain and scale. Accordingly, the biological text-mining field has evolved to identify and extract information from the literature for database storage and access. Two types of tasks predominate in biological text mining: the extraction of gene and protein names [1–4] and the extraction of interactions between proteins [5–7]. The BioCreAtIvE challenge was [8] focused on name extraction [9] with the additional task of functional annotation [10]. Other text-mining applications focus on hypothesis generation [11], probing protein subcellular localization [12], and pathway discovery [13].

Recent work has also focused on the extraction of protein point mutations from biomedical literature [14–18]. Protein point mutations, the substitution of a wild-type amino acid with an alternate one, can be important to our understanding of protein function, evolutionary relationships, and genetic disorders. From a functional perspective, researchers introduce point mutations into proteins to assay the importance of a particular residue to protein function. Evolution relies upon mutations or polymorphisms in DNA, a mechanism for creating diversity in protein sequences. While the term “mutation” is used to imply deleterious changes, and “polymorphism” means a difference within species, for text-mining purposes we refer to a “point mutation” as a substitution of a different amino acid for the reference amino acid. dbSNP [19] and the Human Gene Mutation Database [20] are two of many databases that catalog point mutations and their downstream effects. These databases are manually curated, which limits the speed of input into the database and the breadth of information represented, but does aid in the incorporation of complex information that is difficult for text-mining tools to parse.

The task of point mutation extraction can be decomposed into two subtasks. First, it is necessary to identify the protein and mutation terms discussed within an article. After these entities are identified, an association must be made between the point mutation and its correct protein of origin. This problem is trivial when a paper discusses a single protein but increasingly complex when multiple proteins are present. In our evaluation of Mutation Graph Bigram (Mutation GraB), we downloaded 589 full-text PDF articles related to the GPCR, tyrosine kinase, and ion channel protein families from PubMed-provided links. Using our dictionary-based protein term identification method, we counted 350 articles out of the total 589 that contained a point mutation that could have belonged to multiple proteins. A few methods for point mutation extraction have been developed. Rebholz-Schuhmann et al. [14] describe a method called MEMA that scans Medline abstracts for mutations. Baker and Witte [16–18] describe a method called Mutation Miner that integrates point mutation extraction into a protein structure visualization application. Our own group has presented MuteXt [15], a point mutation extraction method applied to G protein–coupled receptor (GPCR) and nuclear hormone receptor literature. MEMA and MuteXt use a straightforward dictionary search to identify protein/gene names and a word proximity distance measurement to disambiguate between multiple protein terms. Both methods, while providing a simple and successful method for point mutation extraction, were limited in two areas. First, the word distance measurement is not always correct in disambiguating between protein terms. Second, MEMA was evaluated on a set of abstracts, which are intrinsically more limited than the full-text article. In our literature set, the abstracts contained only 15% of the point mutations found in the full text. The point mutations were also validated against OMIM [21], which only contains disease-related point mutations. MuteXt was trained and evaluated on GPCR and intranuclear hormone receptor literature and contained customizations in the algorithm for dealing with problematic protein naming and amino acid numbering cases.

Mutation Miner approaches the problem differently. This method identifies and relates proteins, organisms, and point mutations using NLP analysis at a sentence level. An entity pair is assigned if both entities match noun phrase patterns. This method would work well if all point mutations were described in conjunction with associated proteins and organisms at the sentence level, which we have observed is not always the case. Mutation Miner also incorporates protein sequence information, but for use in annotating protein 3-D structures with mutation information instead of point mutation validation. Our method improves on MEMA, MuteXt, and Mutation Miner by using a novel graph bigram metric that incorporates frequency and location of terms to disambiguate between proteins and searches full-text information. Like MuteXt, Mutation GraB utilizes the Swiss-Prot protein database [22] for sequence validation, which intrinsically contains more sequence variation than OMIM. We addressed the utility of our application by standardizing the algorithm for all protein families and by evaluating our method on three different protein family literature sets covering 589 articles. More detailed comparisons with MEMA and Mutation Miner are described in the Discussion section.

### Protein Term Identification

For our task of associating point mutations to protein terms, it is not sufficient to minimally tag a protein name in the literature; we must also find its correct gene identifier in a corresponding database. The BioCreAtIvE challenge addressed this problem with the 1B subtask of identifying a protein/gene mentioned in the text and annotating it with its correct gene identifier. Solutions for this challenge ranged from rule-based methods [23] to machine-learning approaches [24] to a combination of both. Unfortunately, some of these methods may not be applicable to our point mutation extraction task. The participants in the BioCreAtIve challenge were provided a large set of annotated sentences categorized under three different organisms; human, yeast, and fly. Some solutions for the subtask 1B consisted of learning the training data for each organism, then applying the learned functions to a test set also divided by organism. This approach is suboptimal for our task for two reasons. First, because point mutations are frequently analyzed at a protein family and superfamily level, methods trained on protein names from organism-specific lexicons would not be well-suited for analysis across many species. Second, our goal is to create a broadly applicable methodology for point mutation extraction that can be utilized on any categorization of proteins (i.e., family, class, fold, etc.). Machine-learning approaches benefit from large detailed annotated training sets. In our experience, the manual labor involved in annotating the amount of text necessary to learn protein family–specific nomenclature on the scale presented by BioCreAtIve is likely to undermine the benefits of automated point mutation extraction.

Methods relying solely on rule-based features for protein-name identification generally perform at a lower precision and recall than methods incorporating machine learning. However, since rule-based methods do not necessarily require annotated training data, they are advantageous when such data is unavailable or difficult to acquire. Our approach to protein term identification is similar to other rule-based approaches [2,23,25]. We first create a dictionary using the names and synonyms of proteins in a protein family; the protein names are retrieved from their respective Swiss-Prot and Entrezgene entries. The terms in the dictionary are then searched for in the journal literature. Depending on the character length and composition of these terms, we search by different regular expressions with varying levels of specificity. A further description of this is detailed in the Methods section.

### Point Mutation Identification

Point mutations are represented in a variety of ways in the literature, but all consist of three distinct parts: a wild-type amino acid, a sequence position, and a mutant amino acid. A typical representation of a point mutation is A123T, denoting a change from alanine to threonine at position 123 of a protein using the single letter abbreviation for the amino acids. Variations on this shorthand form include A123 → T, A(123)T, and A-123-T, and the three-letter amino acid abbreviations Ala123Thr, Ala123 → Thr, Ala(123)Thr, and Ala-123-Thr. Aside from those frequent representations, point mutations are also represented grammatically such as “position 123 was mutated from an alanine to a threonine” or “positions 100–110 were mutated to proline.” In our literature sets, however, <1% of all true positive point mutations were grammatical, so we chose to focus on the single-letter and three-letter abbreviation variants of point mutations instead.

### Point Mutation–Protein Association

The task of associating point mutations to proteins is unique and has no true corollaries from other text-mining applications. Protein–protein interactions are explicit binary relationships that usually occur locally within a sentence or two. A point mutation usually has a one-to-one relationship with a protein; however, this relationship is often implicit over the length of the whole text. For example, a journal article may present a protein term in the abstract and the introduction sections while describing point mutations to that protein in the methods and discussion sections. It is implied that the point mutations discussed in the latter sections refer to the protein term in the former sections. When more than one protein is discussed in the text, a method is required to choose the correct protein or species.

**Figure 1:**
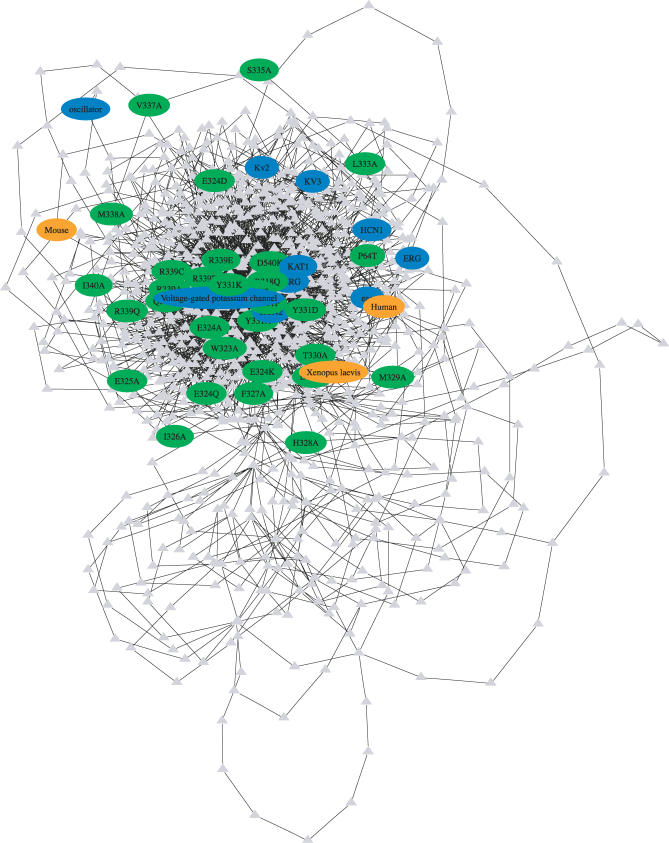
An Energy-Minimized Graph Generated from the Full-Text Article PMID 11553787 The blue ellipses represent protein term nodes, green ellipses represent point mutation nodes, and orange ellipses represent organism nodes. The gray triangles represent regular words. The connecting edges show terms or words represented by the nodes that are present as a bigram in the text. For this article, a total of 1,052 terms are contained in 2,287 bigrams.

Previous methods have used a simple and effective word distance metric for protein term disambiguation; a point mutation is assigned to its nearest occurring protein term. Our graph bigram method improves on this approach by accounting for all occurrences of the point mutation and protein terms throughout the length of the text instead of measuring one local relationship. This method uses the *t* test to measure the significance of bigrams in the text, then employs a graph shortest-distance search to traverse significant bigrams to associate a point mutation with its correct protein term. An example of a graph generated from an ion channel transporter article (PMID 11553787) is shown in Figure 1. While this graph is too complex to provide any algorithmic examples, we can see that nodes found closer to the center grouping in the graph are involved in more bigrams than the peripheral nodes. In general, paths that traverse the central grouping of nodes will be shorter and more significant than paths taken around peripheral nodes.

### Mutation GraB Approach

Our approach to point mutation extraction consists of the following steps. 1) Target a protein family of interest and retrieve full-text articles discussing point mutations within the protein family. 2) Identify protein and organism terms within the articles using a dictionary generated from protein databases (creating an implicit link between the protein term and database identifier). 3) Identify point mutation terms using a set of regular expressions. 4) For each point mutation, generate a set of possible associated proteins by comparing the wild-type amino acid with that contained in the protein sequence. If this set contains several possible proteins, use the graph bigram method to disambiguate and to find the correct association.

**Figure 2:**
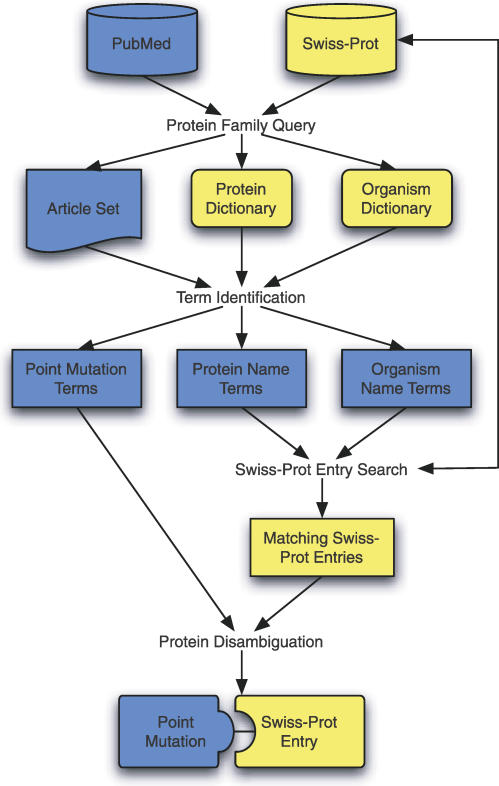
A General Overview of the Process Flow of Mutation GraB

The process flow of Mutation GraB is shown in Figure 2. In our execution and evaluation of Mutation GraB, we wanted to focus on three different aspects of point mutation extraction. First, we wanted to gauge the feasibility of systematically extracting point mutations from the literature in a fully automated fashion. Our initial testing and previous methods showed that automated point mutation extraction is a wholly viable endeavor, with the main challenges of identifying protein terms and associating them to the proper point mutation. Second, and most important, we wanted to assess different search methodologies available to extract point mutations and to devise a superior search metric for extraction. We hypothesize that a search metric that integrates the relative position of the words and frequency data into its heuristic will outperform a metric that solely relies on positional information. Last, we wanted to create an application that could be used extensively on all protein family literature to create a database of point mutations. Therein, Mutation GraB is a self-contained application where most, if not all, the information used is gathered from database sources and not expert-user opinion.

## Results

We evaluated the effectiveness of Mutation GraB by using it to extract point mutations from literature describing three different protein families: tyrosine protein kinases, GPCRs, and transmembrane ion channels. Since most studies of protein structure and function focus at the protein family level, and different protein families have specific and differentiating nomenclatures, this approach is representative of real-life usage and tests the flexibility of Mutation GraB for distinct protein families. Each of the protein family literature sets were split into two groups, a “development” set and a “validation” set of articles. We selected the articles for each set randomly from the all the articles downloaded for each protein family. The development set was used to optimize Mutation GraB performance, while the validation set was used to confirm the performance on the development set. The number of articles in each protein family literature set and the number of true positive mutations manually identified is listed in Table 1, and the Swiss-Prot entry, protein name dictionary, and organism dictionary sizes are listed in Table 2. Throughout our efforts, these datasets were kept entirely distinct so that the validation set represents a true measure of the generality of the algorithm optimized on the development set. Within each protein family literature set, we ran Mutation GraB twice, once using the graph bigram association metric and the second time using the word distance metric. Performance for each search metric can be compared within each protein family literature set.

**Table 1:**
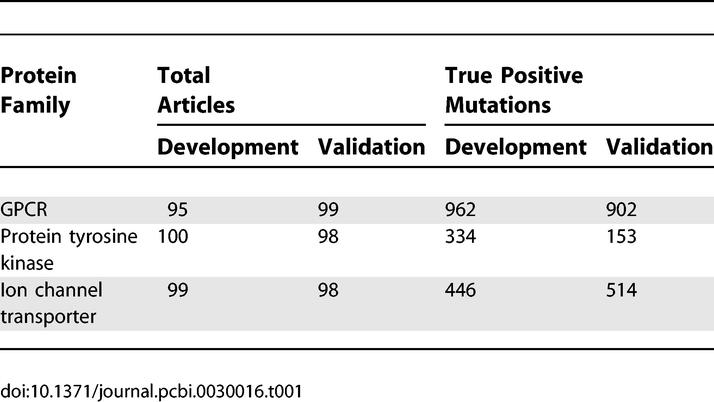
Protein Family Literature Sets

**Table 2:**
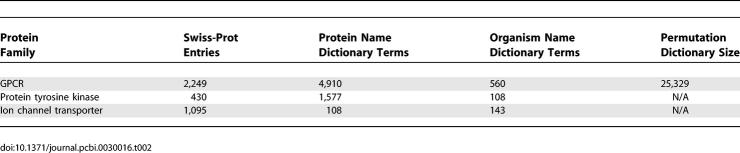
Protein Family and Dictionary Information

### Evaluation Methods

Mutation GraB was scored against manually annotated “gold standard” sets for each protein family (Datasets S1–S6). Point mutations that Mutation GraB and manual curation assigned to the same protein are considered true positive (TP) classifications. Point mutations that Mutation GraB assigned to a protein but were manually classified discordantly are counted as false positive (FP) mutations. In addition, point mutations that were manually classified as TP, but assigned to the wrong protein by Mutation GraB are also ruled as FP mutations. Point mutations that Mutation GraB missed but manual curation assigned are labeled false negative (FN) mutations.

We chose to evaluate Mutation GraB in two different ways. First, we compared the traditional text-mining measurements of precision, recall, and balanced F-measure between the graph bigram and word distance metrics within the development, validation, and complete protein literature sets. Precision is calculated as P = TP / (TP + FP), recall is calculated as R = TP / (TP + FN), and the balanced F-measure is computed as 2*P*R / (P + R). Second, and more significantly, we examined the precision of each search metric on point mutations versus the number of possible protein associations (PPA) for each point mutation. Since the main purpose of the search metric is to disambiguate multiple proteins for each point mutation, the more robust metric will have a higher precision at higher PPA.

### G Protein–Coupled Receptors

For GPCRs, we took advantage of the manually curated tGRAP database [26] to identify journal literature that describes GPCR point mutations. The tGRAP database subset contains a total of 5,451 point mutations, 1,495 GPCRs, and 914 article citations. We retrieved 386 of these citations as PDF documents and annotated 95 articles as a development set and 100 articles as the validation set. The Swiss-Prot database [22] contained 2,249 entries that correspond to GPCR proteins. From these Swiss-Prot entries and their existing corresponding Entrezgene [27] entries, a standard protein name dictionary of 4,910 terms, an organism dictionary of 560 terms, and a protein name permutation dictionary of 25,329 terms were generated. A permutation dictionary is generated by taking protein name terms of three words or greater and changing the order of the words. We observed that the use of both standard and permutation dictionaries was helpful in identifying a greater number of protein names than the use of the standard dictionary alone. We describe the generation of the permutation dictionary in detail within the Methods section.

The performance of the word distance and graph bigram metrics for the development and validation sets are shown in Table 3. In the development set (Dataset S1), the graph bigram metric achieved an F-measure of 0.76, while the word distance metric achieved an F-measure of 0.70. Examining the point mutation counts between the two metrics, we saw that the graph bigram metric was able to identify 636 true positive mutations versus 565 for the word distance metric. In the validation set (Dataset S2), the graph bigram metric and word distance metric achieved F-measures of 0.83 and 0.81 with true positive mutation counts of 684 and 652, respectively. Combining the two sets, the graph bigram metric outperformed the word distance metric with an F-measure of 0.79 to 0.76.

**Table 3:**
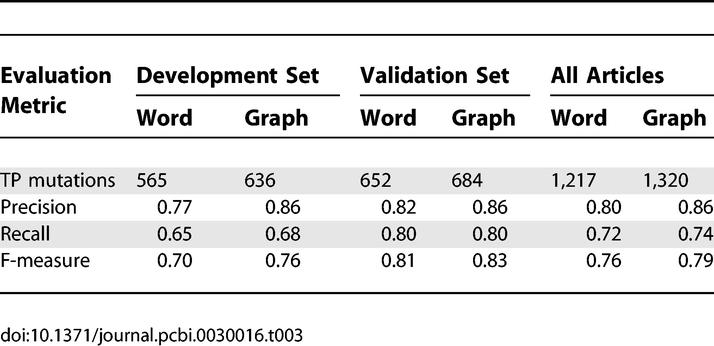
Mutation GraB Performance on the GPCR Literature Sets

**Figure 3:**
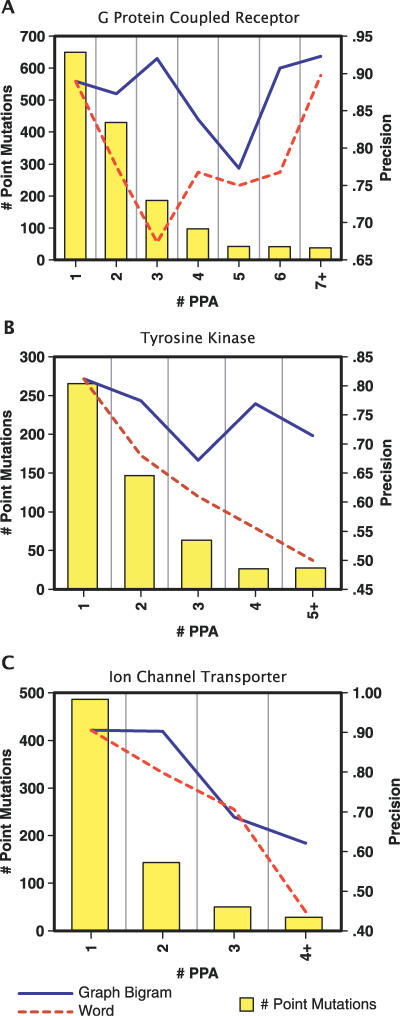
Examining the Precision of the Graph Bigram and Word Distance Metrics across Different Levels of Possible Protein Associations for the GPCR (A), Protein Tyrosine Kinase (B), and Ion Channel Transporter (C) Literature Sets This data is for the cumulative development and validation sets combined. The yellow bars show the number of point mutations counted at each PPA. The solid blue line represents the precision measured for these point mutations using the graph bigram metric, and the dotted red line is measured using the word distance metric.

Figure 3A–3C graphs the precision of both search metrics measured at different PPA levels for all three protein family literature sets. The precision is measured for the development and validation sets together. We cannot calculate the recall for this analysis because false negative point mutations belong to a protein not represented in the possible associations. The yellow bars represent the number of point mutations counted at each level of PPA. For the GPCR literature set shown in Figure 3A, there was a large spread of PPA for point mutations. While 651 point mutations only had one PPA, 844 point mutations had multiple possibilities, and the average number of associations per point mutation was 2.19. Figure 3A shows that the graph bigram metric achieved a higher precision at all levels of PPA greater than one for the GPCR literature sets.

### Protein Tyrosine Kinases

We were able to retrieve 554 PDF articles from the PubMed protein tyrosine kinase query “tyrosine kinase[mh] AND point mutation[mh] AND full text[sb]”. We annotated 99 of these articles for use as our development set and 98 articles as our validation set. Searching the Swiss-Prot database for protein tyrosine kinases yielded 430 different entries. The protein tyrosine kinase standard dictionary contained 1,577 terms, and the organism dictionary contained 108 terms. Our initial tests indicated that the use of a permutation dictionary would hurt performance for this protein family as many protein names have several elements in common with other family members.

Table 4 summarizes our results for the protein tyrosine kinase literature sets. The articles as a whole contained fewer TP and more TN point mutations than the GPCR literature sets, affecting performance by decreasing precision for both graph bigram and word distance metrics. Performance on the development set (Dataset S3) for the graph bigram and word distance metric were closer together, with F-measures of 0.74 and 0.70, respectively. The validation set (Dataset S4) yielded even closer results with F-measures of 0.68 for the graph bigram metric and 0.67 for the word distance metric. This small difference is largely due to the few number of true positive mutations in the validation set, with the graph bigram metric identifying 133 to the 130 identified by the word distance metric. Overall, the graph bigram metric outperformed the word distance metric (F-measure 0.72 versus 0.69).

**Table 4:**
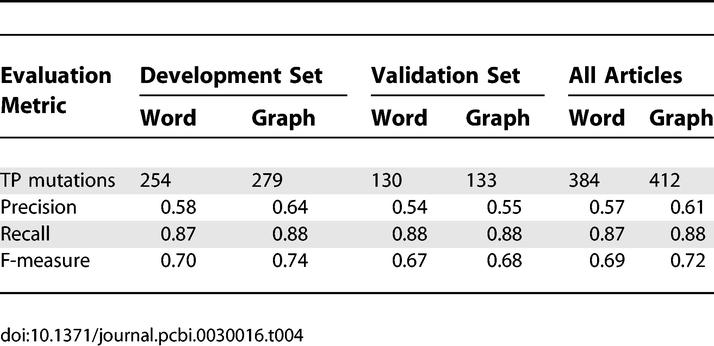
Mutation GraB Performance on the Protein Tyrosine Kinase Literature Sets

The protein tyrosine kinase analysis in Figure 3B shows a smaller distribution of PPA than the GPCR literature set with 266 single PPA, 266 multiple PPA, and a 1.85 PPA average. However, as with the GPCR literature set, the graph bigram metric achieved a higher precision at all PPA greater than one. The greatest difference in precision was for point mutations with four or more PPA where the graph bigram metric had more than a 0.21 increase than the word distance metric.

### Ion Channel Transporters

The ion channel articles were identified with the PubMed query “ion channel[mh] AND point mutation[mh] AND full text[sb]”, and 311 PDF articles were downloaded and converted to text. We used 100 of these articles as the development set and 98 articles as the validation set. A total of 1,095 ion channel proteins were identified using the Swiss-Prot “Ion Channel” and “Transporter” keyword identifiers, and 3,089 protein names were extracted from the associated Swiss-Prot and Entrezgene entries. As with the protein tyrosine kinase literature set, the use of the permutation dictionary did not identify a greater number of protein names, so only the standard protein name dictionary was used. The organism dictionary contained 143 organism names.

Table 5 shows the results of the graph bigram and word distance metrics on the development (Dataset S5) and validation (Dataset S6) sets. Consistent with the GPCR and tyrosine kinase literature sets, the graph bigram metric had a greater performance gain in the development set (F-measure of 0.70 to 0.68) than in the validation set (F-measure of 0.80 versus 0.79). The graph bigram metric outperformed the word distance metric for both datasets, and overall the graph bigram metric achieved a higher F-measure of 0.76 to 0.74, extracting 624 TP mutations to 604 TP mutations for the word distance metric. The ion channel literature sets yielded the smallest performance difference between the two different search metrics.

**Table 5:**
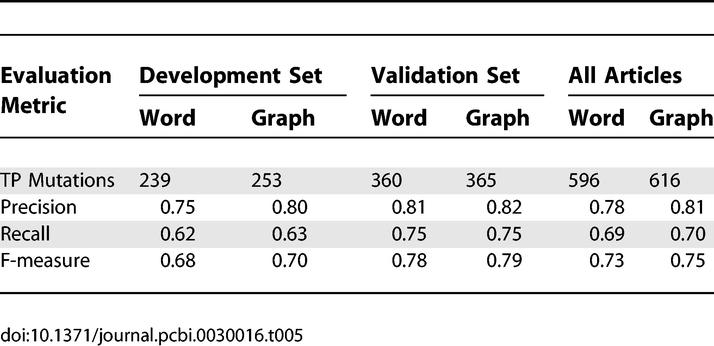
Mutation GraB Performance on the Ion Channel Transporter Literature Sets

Figure 3C shows that the ion channel literature set has the fewest PPA per point mutation out of the three protein families. We counted 487 point mutations with only one PPA, while only 224 point mutations had multiple PPA. The average PPA per point mutation is also the smallest at 1.53. The precision difference measured across different PPA levels is less pronounced in the ion channel transporter literature set, with the word distance metric outperforming the graph bigram metric with a precision of 0.71 to 0.69 on point mutations with three PPA. At two PPA and four or more PPA, the graph bigram metric still achieves a higher precision than the word distance metric.

## Discussion

We have introduced Mutation GraB, an application for identifying and extracting point mutations from biomedical literature. Our goal with Mutation GraB was to create a general-purpose application that could have consistent performance without relying on protein family customization. Across these representative protein families, customization was not required to achieve consistently accurate performance in both development and validation sets. This suggests that Mutation GraB should be useful for identifying point mutations in most if not all protein families. Mutation GraB performance is contingent on a number of factors, including the identification of protein names, organism names, and point mutation terms in the text, and the disambiguation of multiple proteins when present. We chose a rule-based approach for protein name identification that has been shown to be successful in other tests, and the search for organism names is accomplished with straightforward pattern matching. Our main performance goal, however, was to devise a disambiguation metric that outperforms current methods at choosing the correct protein from a selection of several possible choices.

### Comparison with MEMA

MEMA and Mutation GraB were created and tested in a different fashion that makes a direct comparison troublesome. MEMA was tested on 16,728 abstracts across many protein families with the precision and recall estimated from a random set of 100, while we have chosen to use full-text articles from selected families and provide the precision and recall for all 589 articles. The point mutations extracted by MEMA were associated with proteins contained in HUGO and validated with mutations in OMIM, while Mutation GraB utilizes the Swiss-Prot and Entrezgene databases for protein identification and sequence validation. MEMA also extracted DNA mutations from their set of abstracts, and while the current version Mutation GraB can identify both DNA and protein point mutations, we only validate protein point mutations. Additionally, MEMA identifies a wider set of mutation types, including some that are described grammatically. Table 6 shows the performance of Mutation GraB against that of MEMA on the set of 100 abstracts with these caveats.

**Table 6:**
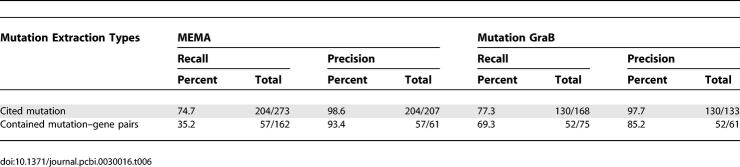
Mutation GraB versus MEMA Performance

The row “Cited mutation” refers to the identification of the point mutation terms in the text, while the row “Contained mutation–gene pairs” refers to the identification and association of the mutation to its protein of origin. The different counts are because of the absence of DNA mutations in the Mutation GraB analysis. Also, our manual analysis of the abstracts found a few mentions of mutated amino acid positions without specifying a mutant amino acid. These mentions were not included in our counts. The precision and recall for identifying “cited mutation” are essentially the same for both MEMA and Mutation GraB. Considering that Mutation GraB does not identify any grammar mutations while MEMA does, this is somewhat surprising. In comparing the identification of “contained mutation–gene pairs,” however, Mutation GraB achieves a much higher recall (77.3% versus 35.2%) but a lower precision (85.2% versus 93.4%) than published results for MEMA. As Mutation GraB validates the mutation–protein pairs by comparing with Swiss-Prot sequences, these associations are more significant and may contribute to a lower number of total mutation–gene pairs found in the text. Mutation GraB's disambiguation metric and sequence validation steps help decrease the number of incorrect associations, thereby increasing the recall significantly.

In addition to the increase in recall, we believe Mutation GraB to be an improvement over MEMA for other reasons. One, while abstracts are more readily available than full-text articles, full-text articles are far more informative with regard to point mutations. MEMA extracted 24,351 point mutations mentions from 16,728 abstracts for an average of 1.45 point mutations per abstract. The 589 articles that Mutation GraB was evaluated against contained 3,216 unique point mutations, resulting in an average of 5.45 point mutations per article. Because Mutation GraB only counts unique point mutations per article, the total number of point mutation terms identified is actually significantly higher. We found that a full-text article contains approximately seven times more point mutation mentions than the abstract alone. With this in mind, our processing of 589 full-text articles would be equivalent to a larger quantity of abstracts.

Second, validation against OMIM will only compare point mutations that are disease-related, while sequence validation against Swiss-Prot compares all point mutations that differ from the wild-type amino acid. Since Swiss-Prot is updated more frequently and contains more genes/proteins than OMIM, Mutation GraB can validate a greater number of point mutations than MEMA. Finally, out of the 100 abstracts MEMA analyzed, only 35 contained multiple gene/protein mentions. From our set of full-text articles, 81 contained point mutations belonging to multiple proteins, and we counted multiple gene/protein mentions in 562 out of 589 articles (95%). As a result, the protein disambiguation capability of Mutation GraB was tested more rigorously than MEMA.

### Comparison with Mutation Miner

Mutation Miner differs in many respects to Mutation GraB. Foremost, their use of NLP on a sentence level differs from the statistical approach of Mutation GraB. When searching for a protein of origin, Mutation Miner uses the protein and organism terms to query Entrezgene for a unique protein entry to retrieve sequence information. They show this solution to be suboptimal, because multiple proteins may be retrieved from the query and sometimes the target protein is not the first protein returned. Also, Mutation Miner uses the protein sequence information not to validate extracted point mutations, but instead to produce multiple sequence alignments of targeted proteins to provide mutation annotations to 3-D structure displays.

The authors of Mutation Miner tested their methods on 19 abstract and full-text articles on the xylanase protein family. We sought to run Mutation GraB on the same 19 full-text articles to generate a performance comparison, but ran into some obstacles. Instead of measuring the precision and recall for the correct association of a protein–organism pair with each point mutation, Baker and Witte et al. compute precision and recall for the identification of protein–organism pairs and the identification of point mutations separately. Since Mutation GraB identifies protein–organism pairs based on sequence validation against the point mutation, we cannot produce a comparable evaluation. Additionally, we were only able to retrieve 16 full-text articles from the list. One PDF was copy-protected, while two others did not have the full text available from PubMed. In those instances, the abstract was used. Also, the authors of Mutation Miner have counted a total of 54 point mutations in their 19 articles, while we have manually identified 111 point mutations. This discrepancy may affect the precision and recall of Mutation GraB since we are extracting twice as many point mutations.

Table 7 shows the PMID of the articles tested, format of the text, proteins described, point mutations identified, and numbers of point mutations counted by us (Number PM) and Baker and Witte et al. (MM Number PM). For a majority of the articles, we manually identified more point mutations in the text. Table 8 shows the precison, recall, and F-measure achieved by Mutation Miner in extracting protein–organism pairs and identifying point mutations for these articles. It also shows performance for Mutation GraB on the same article set, save for the three abstracts used. We can see that Mutation GraB is better at identifying point mutations than Mutation Miner, with an F-measure of 0.94 versus 0.90, even though we tested on a larger set of point mutations. Mutation GraB also extracted point mutation–protein–organism triplets at a higher accuracy than Mutation Miner extracted protein–organism pairs alone, with an F-measure of 0.87 versus 0.61. Judging by the low recall of Mutation Miner in extracting protein–organism pairs, analysis at the sentence level misses a majority of the protein–organism associations.

**Table 7:**
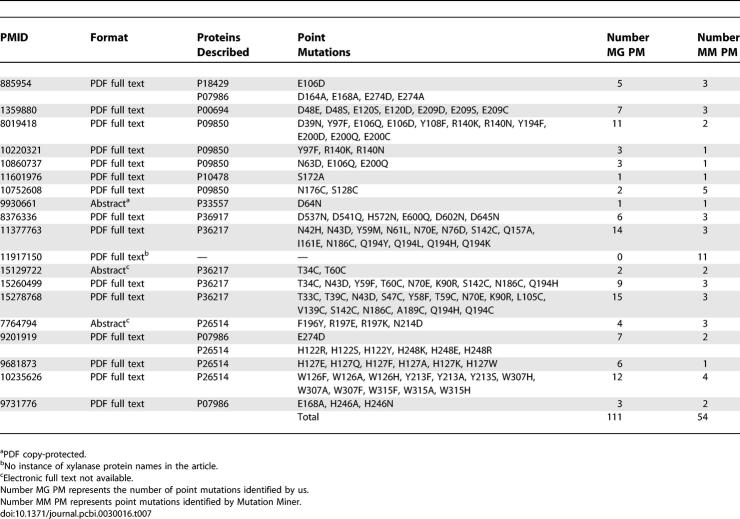
Xylanase Literature Set and Proteins and Point Mutations within

**Table 8:**
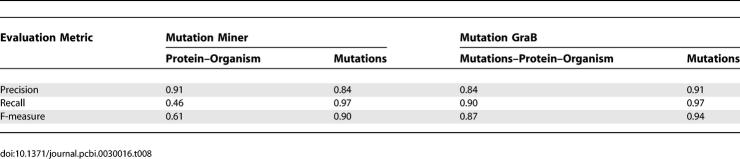
Mutation GraB versus Mutation Miner Performance

### Protein Name Identification

A critical component of point mutation extraction is identifying the protein names for association with the point mutation terms. Since we do not have the luxury of large annotated training sets for our protein families, which are commonly used in more sophisticated methods for protein name recognition and normalization, we relied on a rule-based method. Our rule-based method was patterned after other quantified methods [23,25] and should provide similar performance characteristics. In our set of 589 journal articles, true positive point mutations were represented by 519 proteins and we were able to identify 446 of these proteins for a precision of 0.86. Reasons for missing some of the protein names can be broadly grouped into two categories: (1) difference in name representation from Swiss-Prot or Entrezgene and (2) formatting changes as a result of PDF-to-text conversion.

An example of the first category is the identification of the Scn4a protein (Swiss-Prot AC: P15390), whose synonyms are “Mu-1”, “microI”, “Voltage-gated sodium channel alpha subunit Nav1.4”, “Nav1.4”, “Sodium channel protein type IV alpha subunit”, “NCHVS”, and “Sodium channel protein, skeletal muscle alpha-subunit”, as given by Swiss-Prot and Entrezgene. In the ion channel article PMID 10653790, this same protein is represented by the term “NaCh”, presumably as an abbreviation for “sodium channel”. However, the term “NaCh” is not remotely close to any of the provided synonyms given for the Scn4a protein. Another example is the identification of the protein Q98146 in the GPCR article PMID 10842179. The only synonym given for this protein is “G-protein coupled receptor homolog 74”, and the representation used in the article is ORF74. In both of these instances, our dictionary-based search could not have possibly identified the protein terms in the article with the synonyms at hand.

The PDF-to-text conversion of journal articles also often generates unintended changes with regard to protein names. One such consequence is the modification of superscript and subscript formatting present in some PDF files.

Another effect of the PDF-to-text conversion is the mishandling of Greek characters. The pdftotext utility replaces Greek characters with their Unicode representation, and unless the characters are represented in Unicode within the PDF, the conversion removes them. Many protein names, especially in the GPCR family, rely on these designations for differentiation from other similar proteins. While the Unicode representation is found in some PDF files, frequently other font or image representations are used to denote Greek characters. When the non-Unicode Greek characters are removed from these names, they are either skipped or misidentified for other terms. The ion channel article PMID 10097182 describes the α, β, and γ ENaC proteins (Swiss-Prot ACs P37089, P37090, and P3791). During the PDF-to-text conversion, these characters were stripped, making it impossible to identify which ENaC proteins are being discussed.

A number of overlooked protein names in the GPCR literature set could be recovered using the permutation dictionary, however. For historical reasons, GPCRs were originally named by physiologists studying features, then by pharmacologists focused on tissue specificity, and finally by the genomics community based on sequence homology. Some GPCRs have been renamed on more than one occasion, and the order of naming elements is often permutated. These factors are less relevant to the ion channel and tyrosine kinase literature; thus, the use of a permutation dictionary increased the recall by identifying some full-length protein terms, but this benefit was limited to the GPCR family. One example where the permutation dictionary was useful is the “Parathyroid Cell calcium-sensing receptor” (Swiss-Prot AC P41180). Protein symbols for this term include “CaSR”, “Gprc2a”, “Pcar1”, and “FHH”; a wide variety of legacy naming. Unfortunately, authors frequently use the term “calcium sensing receptor” to describe this protein. While that term is less specific than the original full name, it is specific enough to identify that single Swiss-Prot entry from the set of GPCR entries. A permutation dictionary helped recover this term while other protein entity recognition methods would probably have not. Owing to the proliferation of GPCRs in the olfactory tissues, the permutation dictionary also contained a large number of nonsensical permutation terms such as “receptor 31 17” generated from the “Olfactory receptor 17–31” term (Swiss-Prot P58170). However, these nonsensical terms are unlikely to be found in the text and the additional cost of precomputing the permutation dictionary and additional searching is modest. The protein tyrosine kinase and ion channel transporter literature, in contrast, did not benefit from the use of the permutation dictionary for identifying additional terms. The literature for these protein families contained a more standardized nomenclature, and the use of the permutation dictionary only increased the number of spurious terms identified.

Since protein term identification is independent from the rest of Mutation GraB, a switch from one method to another is transparent to the other parts. While protein name identification is a necessary component of Mutation GraB, it is not the full focus of our efforts and is more thoughtfully addressed in the recent BioCreative challenge.

### Protein Disambiguation

The main task of the search metrics was to select the correct protein to associate with a point mutation when several proteins are found in the text. When only one protein is found whose sequence matches the point mutation wild-type amino acid, no disambiguation is necessary; the graph bigram and word distance metrics are not utilized. In instances where more than one protein can be assigned to a point mutation, the search metrics are used to disambiguate. Therefore, the main performance difference between the two search metrics is not the overall F-measure, but the precision measured in instances of multiple protein disambiguation.

Figure 3A–3C provides evidence that the graph bigram metric performs better than the word distance metric in these instances. Figure 3A–3B shows that the graph bigram metric achieves a higher *P* at all levels of PPA greater than one, while Figure 3C shows the graph bigram metric ahead in all cases except for three PPA. The GPCR point mutations were ideal for this analysis because of the wide range of PPA values; a large number of mutations had two to six PPA. The protein tyrosine kinase and ion channel transporter literature sets contained fewer point mutations in general and a smaller spread of PPA. The ion channel transporter set, especially, contained more than twice as many point mutations with one PPA >1 PPA. This fact can explain why the overall F-measures between the two metrics for the ion channel transporter literature sets are quite similar. For the set of point mutations with PPA ≥ 1, the precision P = 0.84 using the graph bigram metric and P = 0.73 using the word distance metric. This highlights the value of the graph bigram metric over the word distance metric in disambiguation situations.

**Figure 4:**
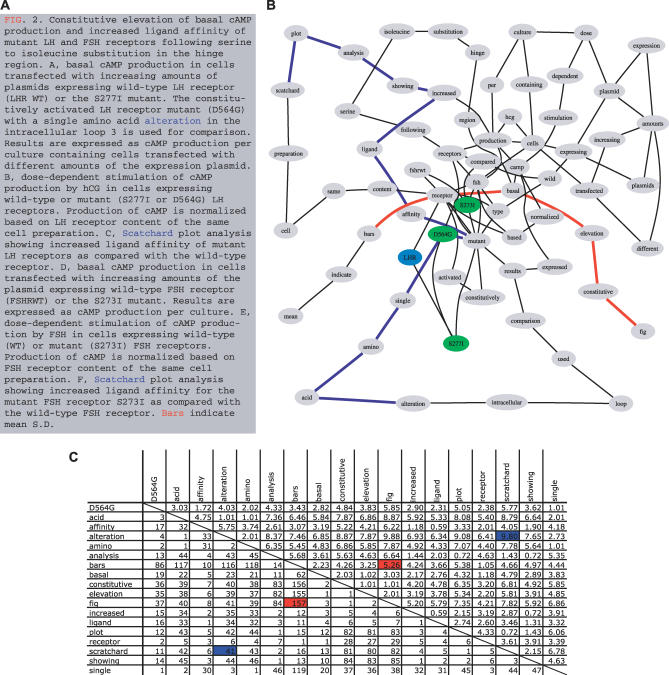
Example of a Paragraph of Text Evaluated by the Graph Bigram and Word Distance Metrics (A)Text is taken from a figure label from the article PMID 10889210. (B)Graph generated by bigram traveral using the graph bigram method. The point mutation terms are in green, protein terms in blue, and regular words in gray. (C)Table shows the measurements between some selected words in the text using both the word distance and graph bigram metrics. The word–distance measurements are below the diagonal, and the graph bigram measurements are above the diagonal. Two different word pairs are examined, {fig, bars} and {alteration, scatchard}. The {fig, bars} words are shown in red in (A), the path is colored in red in (B), and the metric measurements are highlighted in red in (C). The {alteration, scatchard} items are highlighted in blue, correspondingly.

The basic assumption in using a word distance metric for point mutation extraction was that the relative positioning between entities in text is the best barometer of associability and significance. We do know, however, that authors describing point mutations often reference nonassociated proteins in close proximity to point mutations, having referenced the associated protein in a different part of the text. This led us to conjecture that frequency as well as positional data, codified in the graph bigram search metric, would be a better method for associating entities for point mutation extraction. Data from Figures 2–4 with PPA >1 supports this conjecture.

### Manual Point Mutation Annotation

To approximate the performance of Mutation GraB on a large scale of articles with the breadth of PubMed, it is important to develop and test it on a smaller set of representative articles. The size of our algorithm development and validation sets reflects a compromise between what is possible and what is practical. The manual processing time for one article ranges from 10–60 min, depending on the number of point mutations in the article and the difficulty in validating them against the Swiss-Prot database. The definition of these “gold standard” annotations may change with each updated release of Swiss-Prot, as some protein accession numbers, protein names, protein keyword classifications, and organism names change with each release. The generation and updating of the gold standard annotations can take as long as 100 h per 100 article set. This, coupled with the current difficulty in retrieving full-text PDF articles from journal sources, makes it prohibitive to work with literature sets larger than 100 articles. Fortunately, the trends in electronic publishing and the more open dissemination of scientific literature favor the availability of an increasingly large set of full-text articles.

### Point Mutations in Images

When identifying point mutations in an article, we counted mutations that occurred within images as true positive mutations. These point mutations were represented commonly as text that occurs in a graphical diagram or chart. Because the information encapsulated within the image is not accessible to text-mining methods, Mutation GraB cannot extract those mutations if they occur exclusively within images in an article. Since a human reader can still identify those mutations, we felt it necessary to include their presence in our gold standard sets. However, removing them from the gold standard sets can more accurately reflect Mutation GraB's performance on solely textual information. Table 9 shows the precision, recall, and F-measure of the three protein family literature sets, with and without the image mutations, processed by Mutation GraB using the graph bigram metric. As expected, the presence or absence of image mutations only affects the recall because they are classified as either TP or TN by Mutation GraB. The GPCR literature set contained the most image mutations, and removing those mutations from comparison would increase the F-measure from 0.79 to 0.87. The tyrosine kinase and ion channel literature sets contained fewer image mutations, and, accordingly, have smaller gains in F-measure with their removal.

**Table 9:**
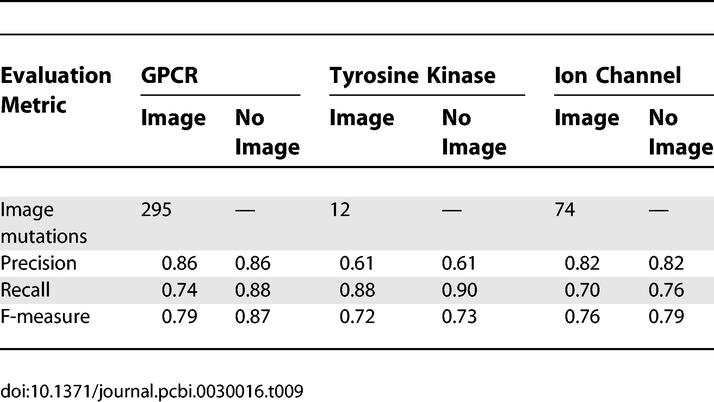
Mutation GraB Performance on All Protein Family Literature Sets with and without Image Mutations Using the Graph Bigram Metric

The GPCR literature set may contain a higher percentage of image mutations because the articles were taken from the tGRAP database and are expected to be more specific on point mutations than its tyrosine kinase and ion channel literature set counterparts. The tyrosine kinase and ion channel literature sets were randomly selected from a resulting PubMed search and have a lower point mutation density due to a lower specificity of subject matter. Nonetheless, when viewing the performance of Mutation GraB on the literature sets, it is important to consider the effect of the image mutations on the recall and overall F-measure.

### Utility

Mutation GraB, if used on a large set of literature, has many potential downstream applications. The immediate benefit would be to generate a database of point mutations found in the literature that could be linked to both its literature and its protein database sources. The result of this database is the ability to examine the effect of point mutations on the structure and function of proteins within the framework of protein families, subgroups, and superfamilies. It is difficult to judge the amount of time saved by using Mutation GraB versus hand annotation, but we estimate this difference as significant. It took upward of 100 h to manually annotate 100 articles, whereas Mutation GraB processed the same volume of articles in about 3 h. Even taking into account hand correction of precision and recall errors, which took anywhere from 10–15 hours per 100 articles, Mutation GraB should still reduce the time required by 80% when compared with exclusively manual annotation. As with most text-mining applications, errors in precision are more tolerable than recall errors; we believe it is more important to identify and label the point mutation, even though the protein association may be incorrect, than to miss point mutations completely. At a F-measure estimate of 0.7, using Mutation GraB and correcting the precision and recall errors is still far more efficient than manual annotation alone. The utility and efficiency of Mutation GraB also relies upon the specificity of the literature given. As one can imagine, examining a very large set of nonspecific articles for a narrow set of protein point mutations will yield low performance.

### Conclusions

From our development and validation of Mutation GraB, we can draw a few conclusions regarding the extraction of point mutations from biomedical literature. Foremost, it is entirely possible to utilize text-mining tools to extract point mutations at a level that warrants its usage. We can process 100 articles in anywhere from 1 h to 3 h, depending on the number of point mutations found within the articles. Also, we know that most articles discuss mutations originating from a single protein. In these instances, no further processing is required to correctly associate mutations and the proteins of origin. For articles that discuss more than one protein, however, a metric for choosing the right protein is necessary. One idea for finding the correct association between proteins and point mutations is to use the word distance between two entities as a metric for association significance. We thought that this metric was insufficient in many regards, and sought to improve it by incorporating frequency data with positional data to generate a heuristic for entity association. The result of this was a metric that combines bigram analysis with graph–theoretic searching that outperforms the simple word distance measure. The graph bigram metric for entity association could have many other applications in the biotext-mining field, and could increase the amount of information that can be automatically extracted from the biomedical literature.

## Materials and Methods

The overview of Mutation GraB is shown in Figure 2. The following sections describe how the protein family literature sets were generated, how Swiss-Prot entries were chosen, and how each article was processed to extract the point mutations.

### Article search and retrieval.

Articles were searched for using PubMed queries containing the protein family name, the MeSH term “point mutation”, and the “full text” filter. The query “<protein family> AND point mutation[mh] AND full text[sb]” was used to retrieve the relevant literature, where <protein family> is substituted by either “protein tyrosine kinase”, or “ion channel transporter”; the GPCR PMID list was retrieved from the tGRAP database. The resulting lists of PMIDs were individually searched using the Entrez E-Utilities, retrieving the LinkOuts—external HTTP links—for the full-text articles. We followed the LinkOuts and parsed the returned HTML pages for links to PDF files, downloading the PDF file when possible. The downloaded PDF files are converted to Unicode text using the Unix “pdftotext” utility.

### Text preprocessing.

After conversion from PDF, the text was preprocessed to create a more cohesive and manageable document. We removed the “References” and “Acknowledgements” sections from the text, as well as sections beginning with the text “this work was supported by”, “to whom correspondence”, and “the abbreviations used are”. A stop list consisting of the words “the”, “and”, “for”, “with”, “were”, “that”, “was”, “from”, “this”, “are”, “which”, “a”, “an”, “or”, and “of” was used and those words subsequently removed.

### Dictionary creation.

Two different dictionaries were created for each protein family to be searched, a protein name dictionary and an organism dictionary. The terms for both dictionaries were extracted from the Swiss-Prot and Entrezgene databases. First, Swiss-Prot entries for the protein families of interest were chosen based on the contents of the “keyword” Swiss-Prot field. Protein tyrosine kinase entries contained the words “Protein Tyrosine Kinase” in the keyword field, GPCR entries contained “G Protein-Coupled Receptor”, and ion channel entries contained both “Ionic channel” and “Transmembrane”. To generate the protein name dictionary, the “protein name” and “gene name” fields from a protein family Swiss-Prot entry subset were compiled. If a Swiss-Prot entry had a related Entrezgene database entry, the “gene name”, “official symbol”, “official fullname”, and “aliases” Entrezgene fields were added to the dictionary if they were not duplicates of the SwissProt names. The organism dictionaries were generated by compiling the “organism” field from the respective Swiss-Prot entries. The word “murine” was added to the organism dictionaries as a synonym for “mouse”.

A second dictionary, which we called a permutation dictionary, was also created from protein full names three words or longer. The entries in this dictionary were permutations of the full names with the permutations also being at least three words long. Since a set of *n* elements will have *n*! permutations, and the number of ways of obtaining an ordered *k* elements from a set of *n* elements is *n*!(*n* − *k*)!, a term that consisted of five words will spawn 5! / (5 − 3)! = 60 permutated terms. For example, permutations of the full name “Parathyroid Cell calcium-sensing receptor” include “calcium sensing receptor”, “cell calcium sensing”, and “cell parathyroid sensing calcium receptor”. Fortunately, nonsensical word permutations are unlikely to appear in actual text. This dictionary is searched in the same manner as the protein full name terms in the standard dictionary.

### Manual annotation of articles.

For each article processed by Mutation GraB, we manually read and extracted the point mutations from the text. We counted a point mutation as a TP point mutation if the associated protein belonged to the corresponding Swiss-Prot protein family set and if the wild-type amino acids of the mutation and the protein matched. A point mutation is considered a TN if its associated protein is not part of the Swiss-Prot set (i.e., belonging to a different protein family) or if the wild-type amino acids do not match. Point mutations that contain typographical errors are considered TN point mutations as well as are point mutations whose position numbering differs from the provided sequence in its corresponding Swiss-Prot entry. These annotated TP and TN point mutations were used as our “gold standard” sets to evaluate Mutation GraB performance. These gold standard datasets are provided in XML format for the GPCR (Datasets S1 and S2), tyrosine kinase (Datasets S3 and S4), and ion channel transporter (Datasets S5 and S6) literature sets.

### Term identification and extraction.

Regular expressions were constructed to identify point mutation, protein name, and organism name terms. A point mutation description usually consists of a wild-type amino acid name, followed by the amino acid position number, which is followed by the mutant amino acid name. The amino acids can be represented in the single-letter or three-letter format, and the regular expressions allow for some punctuation between the position and the amino acids. Some common examples of point mutation strings are “R123Y”, “R(123)Y”, “R-123-Y”, “Arg123Tyr”, “Arg(123)Tyr”, and “Arg-123-Tyr”. Point mutations with a different formatting or representation in the grammar of the text were ignored.

To search for organism names, we created different levels of case-sensitive and insensitive regular expressions. A tiered rule-based approach based on similar methods [2,23,25], however, was used to identify protein name terms. First, the protein name dictionary was split into two groups, symbols (i.e., EPHB1) and full names (i.e., Ephrin type-B receptor 1). Two types of regular expressions were created. One, a strict regular expression, is case-sensitive and does not allow for variation from the protein symbol. A second regular expression, which is more relaxed, is case-insensitive and allows for non-alphanumeric characters to be removed or substituted by spaces. For the protein symbols, both strict and relaxed regular expressions were used with the addition of organism modifier prefixes or suffixes. The prefixes “h”, “m”, and “r” were allowed for human, mouse, and rat modifiers, and the “p” suffix was allowed for S. cerevisiae. For protein full names, both types of regular expressions were used without any additional modifications. We searched with the protein symbols first followed by the protein full names, in each instance using the strict regular expression formation followed by the relaxed. We also allowed for Roman numeral replacement. If a protein name has a single digit as the last character, such as “XYN2”, we also searched for the term “XYNII”.

### Point mutation–protein association.

After identifying all the point mutation, protein name, and organism name terms present in the text, we looked for Swiss-Prot entries that corresponded to the protein and organism names found. For example, the protein full name “Alpha 1-B Adrenergic Receptor” and the organism name “rat” correspond to the unique Swiss-Prot entry P15823. If an article contains multiple protein and organism names, multiple unique Swiss-Prot entries may be represented. The wild-type amino acid of each point mutation was then compared to the amino acid at the specified position of each Swiss-Prot protein found in the text. We also compared the amino acid sequence of any isoforms of the Swiss-Prot protein as well as removing the signal sequence of the protein if present. If the amino acids from the point mutation and the protein sequence match, that protein was categorized as a possible association for the point mutation. When a single Swiss-Prot protein was possible for a point mutation, that protein was automatically associated with the point mutation. When multiple proteins are possible for a point mutation, the word distance or graph bigram methods were used to select the best match for association.

**Word distance metric.** Let **M** be the set of point mutations with multiple possible Swiss-Prot protein associations in an article. For each *m* ∈ **M**, let **P** be the set of protein names and **O** be the set of organism names represented by the possible proteins for *m*. Thus, for *m* ∈ **M**, *p* ∈ **P**|*m*, and *o* ∈ **O**|*m*, we created a list **T** = {*t*_1_ = 〈*m*, *p*_1_, *o*_1_〉, ..., *t*_i_ = 〈*m*, *p*_j_, *o*_k_〉} that represents the PPAs for point mutation *m*. The word distance metric between terms *w*_i_ and *w*_j_ in the text, *h*_word_(*w*_i_ *, w*_j_), is the shortest number of words that separate any two instances of *w*_i_ and *w*_j_. Using this measurement, we associated point mutation *m* to the protein whose protein name *p* and organism name *o* resulted in the smallest δ*_m_*_, word_ = *h*_word_(*m, p*) + *h*_word_(*m, o*) + *h*_word_(*p, o*). This is essentially the triangulation of distances between the point mutation term, protein name, and organism name, where the smallest sum of distances represents the assumed correct association between point mutation and Swiss-Prot protein.

**Graph bigram metric.** The graph bigram metric works in the same manner as the word distance metric in terms of triangulating the smallest distances between the relevant terms in the text. The difference lies in how the distances are calculated. A graph was constructed by assigning nodes to all of the words and terms in the text. An edge connected two nodes if the represented words and/or terms were adjacent to each other in the text. The reciprocal *t* statistic provided a sensible way of measuring how likely it is that any two words will occur next to each other. The *t* test quantifies the likelihood that the adjacency of two words is significant. The larger the *t* statistic, the greater the significance of the relationship. We set the value of an edge between two nodes containing words *w*_i_ and *w*_j_ to be the reciprocal of the *t* statistic:

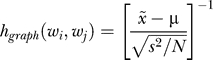
where 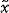 = sample mean, s^2^ = sample variance, N = sample size, and μ = mean of distribution. When the *t* statistic is applied to a text mining application, 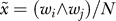 , where 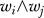 equals the number of times *w*_i_ is adjacent to *w*_j_, and *N* equals the number of words in the text. The mean of the distribution, or the null hypothesis, is *μ* = *w_i_* / *N* × *w_j_* / *N,* where w*_i_* and w*_j_* are the number of occurrences of word *i* and *j*, respectively. For large samples, the variance 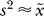 Dijkstra's algorithm is used to calculate the shortest path between any two nodes in a graph, utilizing *h*_graph_ as the edge weight values. Since Dijkstra's algorithm does not work for negative distances, if any *t* statistic for a bigram is negative, all *t* statistics for bigrams in that article are normalized by the negative value so that the smallest *t* statistic = 0. When calculating the δ value for two terms, if the terms are adjacent, we use the reciprocal *t* statistic value. If the terms are not adjacent to each other in the text, we find the shortest path between the two terms, and δ is equal to the sum of distances *h*_graph_ between nodes in the graph within the shortest path.

A more detailed example of the differences between the graph bigram and word metrics is shown in Figure 4. Figure 4A shows text from a GPCR article (PMID 10889210) that was used to create the graph shown in Figure 4B. The text is a paragraph from a figure label from the article. Figure 4C shows the distances generated by the two search metrics between some selected words from the text; below the diagonal the numbers are generated by the word distance metric, and above the diagonal by the graph bigram metric. This example is not meant to show an instance of mutation extraction, but is only meant to highlight characteristics of text that are interpreted differently by each metric. Since most full-text articles have point mutations, protein names, and organism names scattered about the entirety of the text, an example detailing a point mutation extraction would be too complicated to illustrate in a figure. The path in Figure 4B highlighted in red shows a bigram traversal between the word “fig” and the word “bars”. In the text in Figure 4A, we can see that the words are each found only once in the text and they are on opposite ends of the paragraph. Using the word distance metric, *h*_word_(fig, bars) = 157, which is one of the largest distances measured for that text. The graph bigram metric measures *h*_graph_(fig, bars) = 5.26, which, when compared with the other values for *h*_graph_, is not the largest measured. This is because the bigram path traverses the word “receptor”, which is found as a bigram with both “basal” and “bars”. This example shows how two words can be far apart in word distance but still be measured more significantly using the graph bigram metric.

Conversely, we can examine the path in Figure 4B highlighted in blue. The words “alteration” and “scatchard”, when measured by the word distance metric, yield *h*_word_(alteration, scatchard) = 41, meaning there are only 40 words that separate the two. This measure is fairly significant when compared with the other *h*_word_ measurements. However, when using the graph bigram metric, we see that *h*_graph_(alteration, scatchard) = 9.80, a larger and far less significant relationship when compared with other *h*_graph_ measurements. The path generated in the graph for these words is far longer than for “fig” and “bars”, and, accordingly, the graph bigram distance is larger. This highlights a situation where two words close in word distance have a less significant graph bigram measurement.

## Supporting Information

Dataset S1 G Protein–Coupled Receptor Development (1.5 MB XML)

Dataset S2 G Protein–Coupled Receptor Validation Set (1.7 MB XML)

Dataset S3 Tyrosine Kinase Development Set (894 KB XML)

Dataset S4 Tyrosine Kinase Validation Set (650 KB XML)

Dataset S5 Ion Channel Transporter Development Set (1.2 MB XML)

Dataset S6 Ion Channel Transporter Validation Set (1.4 MB XML)

## Abbreviations

GPCR: G protein–coupled receptor
Mutation GraB: Mutation Graph Bigram
PPA: possible protein associations
